# Imaging Hyperreflective Foci as an Inflammatory Biomarker after Anti-VEGF Treatment in Neovascular Age-Related Macular Degeneration Patients with Optical Coherence Tomography Angiography

**DOI:** 10.1155/2021/6648191

**Published:** 2021-02-03

**Authors:** Jing Wu, Chaoyang Zhang, Qian Yang, Hai Xie, Jingting Zhang, Qinghua Qiu, Kun Liu, Dawei Luo, Fang Liu, Jingfa Zhang

**Affiliations:** ^1^Department of Ophthalmology, Shanghai Tenth People's Hospital, Tongji University School of Medicine, Shanghai, China; ^2^Department of Ophthalmology, Shanghai General Hospital (Shanghai First People's Hospital), Shanghai Jiao Tong University, Shanghai, China; ^3^National Clinical Research Center for Eye Diseases, Shanghai Key Laboratory of Ocular Fundus Diseases, Shanghai Engineering Center for Visual Science and Photomedicine, Shanghai Engineering Center for Precise Diagnosis and Treatment of Eye Diseases, Shanghai, China; ^4^Tongji Eye Institute, Tongji University School of Medicine, Shanghai, China

## Abstract

**Purpose:**

To investigate the hyperreflective foci (HRF) as an inflammatory biomarker using optical coherence tomography angiography (OCTA) in neovascular age-related macular degeneration (AMD) patients after antivascular endothelial growth factor (anti-VEGF) treatment and its association with the retinal microcapillary density.

**Methods:**

Twenty-five eyes from 25 patients with neovascular AMD were included in the study. All eyes were imaged with OCTA at baseline (M0) and after 3 consecutive injections (M3; injection performed each month) of anti-VEGF. The number of HRF in the superficial capillary plexus (SCP), deep capillary plexus (DCP), and outer retina was counted. The vascular density of the fovea, parafovea, and the whole macula, as well as the area of the foveal avascular zone (FAZ), was measured.

**Results:**

The mean interval between baseline and follow-up with OCTA was 93.08 ± 5.00 (range, 85-101) days. Compared with the baseline, the number of HRF significantly decreased in DCP (7.52 ± 3.06 vs. 3.76 ± 1.48, *P* < 0.01) and outer retina (12.04 ± 4.91 vs. 5.88 ± 3.32, *P* < 0.01) after treatment. There was no significant difference for HRF number in the SCP, the vascular density (containing foveal, parafoveal, and whole macular), and FAZ area before and after treatments.

**Conclusion:**

The number of HRF in DCP and outer retina might serve as an inflammatory biomarker in patients with neovascular AMD. The reduced HRF possibly represents the alleviation of inflammation after anti-VEGF treatment in patients with AMD.

## 1. Introduction

Age-related macular degeneration (AMD) is the main cause of irreversible severe vision loss in elderly individuals, with a prevalence that exponentially increases with aging. It has been estimated that about 288 million people worldwide will suffer from AMD by 2040 [1]. AMD is subdivided into two types, dry and wet form. The wet form is also called neovascular AMD, which largely benefits from anti-VEGF reagent intravitreal injection. Although the exact pathophysiological mechanism of AMD remains unclear, the prolonged inflammatory response has a crucial role in the onset and development of AMD [2]. During AMD progression, retinal inflammation can lead to the disturbance of the retinal microenvironment, resulting in retinal pigment epithelial (RPE) dysfunction, photoreceptor apoptosis, and choroidal neovascularization [3].

With the advancement of noninvasive retinal imaging technologies, such as optical coherence tomography (OCT), the hyperreflective foci (HRF) can be imaged, which attracted the researchers' attention [4, 5]. HRF in the retina detected with OCT were defined as a discrete and well-circumscribed dot-shaped lesion of equal or higher reflectivity than the RPE band, without back shadowing, and the maximum diameter between 20 and 50 *μ*m [6, 7]. HRF were first described by Coscas et al. in 2009 as the hyperreflective dots (HRD) using spectral-domain OCT in AMD patients [8]. Subsequently, HRF have been reported in many retinal diseases, including diabetic retinopathy, choroideremia, and retinal vein occlusion [9–11]. Although there is still no consensus on its origin, HRF likely characterize a progressive state of the damaged retinal tissue within an inflammatory retinal microenvironment.

Nowadays, many studies that support the hypothesis that activated microglial cells induced by an inflammatory response in the retina are a pivotal origination of HRF [12, 13]. Microglial cells are the major resident immune cells in the retina, which have a critical role in the inflammatory response of neurodegenerative diseases, and are mainly scattered within the inner retina under physiological conditions. Once retinal homeostasis is disturbed, microglial cells proliferate and become activated, releasing proinflammatory cytokines and migrating from the inner retina to the outer retina, thus forming aggregates [14, 15]. These microglial aggregates have been identified as HRF within retinal layers in pathologic conditions, leading to microglia-mediated retinal damage and neuroinflammation. Thus, HRF might reflect a dynamic process of retinal inflammation in different retinal diseases.

In this study, we investigated whether HRF could serve as a possible biomarker of retinal inflammation in patients with neovascular AMD and its possible association with retinal microcapillary density. The number of HRF, vascular density in different retinal layers, and the foveal avascular zone (FAZ) area was measured and quantified with OCT angiography (OCTA) in neovascular AMD patients before and after 3 consecutive anti-VEGF injections.

## 2. Materials and Methods

## 3. Patients

In this retrospective study, we enrolled 25 consecutive treatment-naïve eyes from 25 neovascular AMD patients who were diagnosed with comprehensive ophthalmologic examinations in the Department of Ophthalmology, Shanghai General Hospital affiliated to Shanghai Jiao Tong University, Shanghai, China, between January 1, 2020, and July 30, 2020. The participants with three consecutive monthly intravitreal injections of anti-VEGF agents were enrolled. This study was approved by the Clinical Research Ethical Committee of Shanghai General Hospital affiliated to Shanghai Jiao Tong University (Permits No. 2020KY205) and adhered to the principles of the Declaration of Helsinki. All individual participants provided written informed consent.

The exclusion criteria were as follows: (a) previous treatments for neovascular AMD (including other intravitreal anti-VEGF injections and laser photocoagulation); (b) other vitreoretinal diseases, including dry AMD, vitreous hemorrhage, subretinal fibrosis, and retinal vascular disease; (c) high myopia (refractive errors > 6 diopters of spherical equivalent refraction or >3 diopters of astigmatism); and (d) history of ocular surgery, uveitis, and apparent media opacification.

All participants underwent full ophthalmologic examinations, including best-corrected visual acuity (BCVA), intraocular pressure (IOP) measurement, and anterior segment evaluation using slit-lamp biomicroscopy, fundus photography, and OCTA imaging at the baseline (M0) and 1 week after treatment (M3).

### 3.1. Intravitreal Injection of Anti-VEGF Agent

One experienced surgeon performed all intravitreal injections. The intravitreal injection was performed at the temporal limbus through the eyeball's pars plana under aseptic conditions. All patients received three once-monthly intravitreal injections of aflibercept (2 mg/0.05 mL) using a 30-gauge needle. A 1-week variation was allowed for every injection interval.

### 3.2. Optical Coherence Tomography Angiography (OCTA) Evaluation

**Figure 1:**
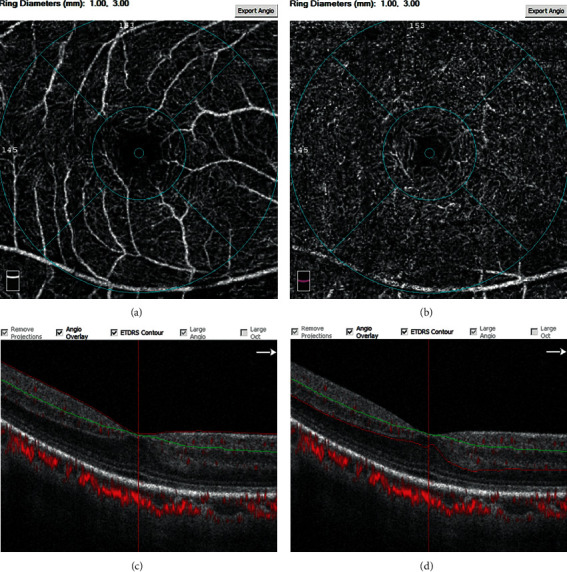
Representative OCT-A images at the 3 × 3 mm^2^ sections centered on the fovea showing the segmentation of microvascular density analysis of the SCP (a, c) and DCP (b, d). As discussed in Section 2, segmentation in the OCT B-scan was set in OCTA automatically. SCP (c), the segmentation was between internal limiting membrane (red line) and 15 *μ*m below IPL (green line); DCP (d), the segmentation was between 15 *μ*m below IPL (green line) and 70 *μ*m below IPL (red line); the segmentation beneath the red line was outer retina (d). DCP: deep retinal capillary plexus; IPL: inner plexiform layer; SCP: superficial retinal capillary plexus.

Retinal microvasculature was imaged using the RTVue XR Avanti OCT system (Optovue, Inc., Fremont, CA, USA), and measurements were acquired using the manufacturer's AngioVue software. The scan covered an area of 3 × 3 mm^2^ sections centered on the fovea. Enface images of the superficial capillary plexus (SCP), deep capillary plexus (DCP), and FAZ area were automatically recorded and analyzed by the OCTA autosegmentation software. The SCP was segmented as 3 *μ*m below the internal limiting membrane and 15 *μ*m below the inner plexiform layer (IPL); the DCP was set between 15 *μ*m and 70 *μ*m beneath IPL, while the outer retina was located beneath the DCP. In the SCP and DCP, two central circles divided capillary plexus into the “fovea area” (a disc with a 0.6 mm diameter) and “parafoveal area” (an internal annular zone, 0.3-1.5 mm from the fovea) as shown in Figure 1. The boundary of FAZ was set by a combination of a canny edge detection algorithm and a level set algorithm centered on the fovea in the whole retinal vasculature [16]. We further manually refined and adjusted the boundary in the case of imprecise segmentation.

The number of HRF was manually counted within a 3 mm diameter centered on the foveal area using a fovea-spanning horizontal B-scan. HRF were subdivided into 3 categories according to different retinal layers: the SCP, the DCP, and the outer retina, as described above. The maximal diameter of HRF was limited within the 20 *μ*m to 50 *μ*m range in order to exclude small counting noise signals as HRF noise signals and prevent large hyperreflective clumps, which are presented as typical hard exudates in fundus photography. Poor quality images with a signal strength index < 4/10 were excluded from further analysis. The quantification of HRF was carried out by two experienced physicians independently.

### 3.3. Statistical Analyses

The data were analyzed by using SPSS 22.0 software. All values are presented as a number or mean ± standard deviation. The visual acuity was presented as the logarithm of the minimum angle of resolution (logMAR). A paired *t*-test was used to compare the number of HRF, the vascular density of SCP and DCP, and the FAZ area between the baseline (M0) and after 3 consecutive monthly anti-VEGF injections (M3). *P* value <0.05 was considered as statistical significance.

## 4. Results

### 4.1. Patient Characteristics

Twenty-five eyes from 25 patients (10 females and 15 males) with neovascular AMD were consecutively enrolled in the study, as shown in Table 1. All patients underwent 3 consecutive monthly injections (M3) of aflibercept. The mean age of patients was 71.4 ± 9.66 years old. The mean interval between baseline and final follow-up was 93.08 ± 5.0 (range, 85-101) days. There was a significant improvement of BCVA from baseline (M0, 0.84 ± 0.41) to the final follow-up (M3, 0.57 ± 0.37), and the mean change of BCVA was −0.25 ± 0.23.

### 4.2. Comparison of HRF in the SCP, DCP, and Outer Retina between Baseline and after the Intravitreal Anti-VEGF Injections

**Figure 2:**
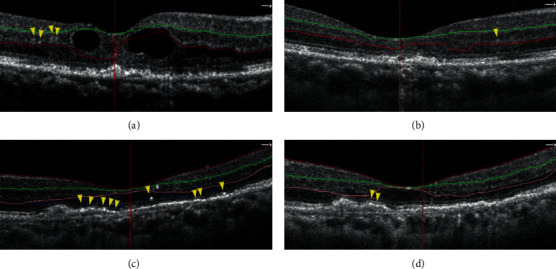
Representative images of two patients showing the HRF in the DCP and outer retina at baseline and after three monthly intravitreal anti-VEGF injections. Patient 1: 63-year-old man with neovascular AMD, HRF were observed in DCP (between red and green line) at the 3 × 3 mm^2^ sections centered on the fovea at the baseline (a) and after anti-VEGF injections (b). The number of HRF was reduced in DCP, and his BCVA was improved from logMAR 1.0 (baseline) to logMAR 0.7 (after treatment). Patient 2: 74-year-old female with neovascular AMD, HRF were observed mainly in the outer retina (beneath the pink line) at the 3 × 3 mm^2^ sections centered on the fovea at the baseline (c) and after anti-VEGF injections (d). The number of HRF was reduced in the outer retina, and the BCVA was improved from logMAR 0.8 (baseline) to logMAR 0.4 (after treatment). The yellow arrowhead indicates the HRF. The microstructures, including the outer limiting membrane, ellipsoid zone, and interdigital zone/retinal pigment epithelial layer, were partially restored in patients after 3 consecutive anti-VEGF treatments.

Analysis of the OCTA parameters revealed that the HRF was distributed across all retinal layers, including SCP, DCP, and outer retina, in neovascular AMD patients. As shown in Table 1, the number of HRF was the highest in the outer retina (12.04 ± 4.91) and the lowest in SCP (5.80 ± 1.63) in the treatment-naïve patient. After the intravitreal anti-VEGF injections, the number of HRF was significantly reduced in the DCP (7.52 ± 3.06 vs. 3.76 ± 1.48, *P* < 0.01) and outer retina (12.04 ± 4.91 vs. 5.88 ± 3.32, *P* < 0.01), compared to baseline; the representative images of HRF were shown in Figure 2. The mean change of HRF in the DCP and outer retinal was −3.76 ± 2.71 and −6.16 ± 4.03, respectively. However, there was no significant difference for HRF number in the SCP before and after treatment (*P* = 0.12).

### 4.3. Comparison of Microvascular Density in the SCP and DCP between Baseline and after the Intravitreal Anti-VEGF Injections

Regarding retinal microvascular density in the SCP and DCP analyzed with OCTA, no significant difference was found for the microvascular density in the foveal, parafoveal, and whole macular sectors before and after treatment as shown in Table 2.

### 4.4. Comparison of FAZ Areas between Baseline and after the Intravitreal Anti-VEGF Injections

**Figure 3:**
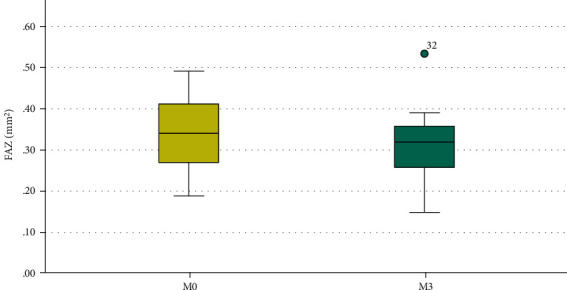
Comparison of FAZ areas between baseline and after the intravitreal anti-VEGF injections. The FAZ areas of the neovascular AMD patients were 0.34 ± 0.09 mm^2^ (M0, baseline) and 0.31 ± 0.08 mm^2^ (M3, after treatment) (*P* = 0.11). Data are shown as mean ± standard deviation.

The distribution of FAZ areas in the retina is depicted in Figure 3. The FAZ areas (mean ± SD) at baseline and after the intravitreal anti-VEGF injections were 0.34 ± 0.09 mm^2^ (M0, baseline) and 0.31 ± 0.08 mm^2^ (M3, after treatment), respectively, with no statistically significant differences (*P* = 0.11).

## 5. Discussion

Microglial cells are considered to have a vital role in the inflammatory response in the pathogenesis of AMD [2, 17, 18]. However, whether microglia are affected by anti-VEGF treatment in AMD patients remains unclear. In this study, we compared the change of HRF numbers before and after anti-VEGF treatment. Our results revealed that the number of HRF in the retina was reduced in DCP and outer retina after 3 monthly consecutive anti-VEGF treatments in patients with neovascular AMD, while the microvascular densities and the FAZ area remained unchanged.

The introduction of high-resolution OCTA has facilitated the study of different capillary plexus in vivo. Previous studies have suggested that HRF may be used as a marker of inflammation in AMD patients [19, 20]. It has been hypothesized that HRF appear after microglial activation and inflammatory cytokines release. Microglial cells are the major resident immune cells in the central nervous system and retina. During physiological condition, the resting microglia are mainly scattered within the inner retinal layers, including the nerve fiber layer, ganglion cell layer, inner plexiform layer, and inner nuclear layer. However, under pathological conditions, activated microglia proliferate and migrate from the inner to the outer retina, secreting proinflammatory cytokines to damage the surrounding retinal neuronal cells [21]. At the same time, activated microglia undergo morphological changes and aggregate to form HRF as imaged with OCT or OCTA.

In our study, the HRF was localized in almost every retinal layer, especially the outer retina of patients with neovascular AMD, indicating the migration and aggregation of microglia cells in the outer retina. Our results were consistent with the previous observations [10, 22], which suggested that HRF might serve as an inflammation OCTA biomarker in neovascular AMD. After aflibercept injections, the number of HRF in DCP and outer retina was significantly reduced, while visual acuity was improved, which might be due to the anti-inflammatory effect of aflibercept. Inflammation is involved in the pathogenesis of neurovascular AMD. This process involves inflammatory cells, such as microglia and monocyte/macrophage, and the related inflammatory cytokines, such as vascular endothelial growth factor A (VEGF-A), placental growth factor (PGF), and monocyte chemokine protein (MCP-1) [23]. PGF and VEGF contribute to the neovascularization by binding VEGF receptor 1 and 2 (VEGFR-1 and VEGFR-2) on endothelial cells, as well as the inflammatory process binding VEGFR-1 on microglia and monocyte/macrophage [24–26]. Besides having an antiangiogenesis function, aflibercept targets VEGF-A/B and PGF, deactivates the inflammatory cells, and reduces the release of corresponding inflammatory cytokines [27]. Therefore, the decreased HRF number and alleviation of inflammation might improve the retinal microstructure on OCTA and the visual function, as indicated by BCVA.

Previous experimental studies have demonstrated that microglia-induced retinal inflammation is a relatively early process during AMD development, which precedes neovascularization, microvascular dysfunction, and retinal neurodegeneration [28]. In this study, we detected the microvascular densities in the foveal, parafoveal, and whole macula of SCP and DCP with OCTA to explore the correlation between HRF number and macular microvascular densities. However, we found no significant differences in vascular densities of the SCP and DCP before and after treatments. The FAZ area represents the level of local retinal ischemia and atrophic changes in macular capillaries. We found no difference in the FAZ area before and after treatments. Therefore, the HRF reduction in DCP and outer retina is likely to be more sensitive and more rapidly responding than the change of microvascular densities in the retina with neovascular AMD after anti-VEGF treatment.

Previous studies have also indicated that HRF could be an inflammatory hallmark, contributing to retinal dysfunction in many retinal diseases. For example, in neovascular diabetic retinopathy, HRF was significantly higher in diabetic patients compared with healthy controls [12]. Furthermore, as inflammatory cytokines could increase vascular permeability in patients with diabetic macular edema, Stela et al. found significant decreases in HRF and central retinal thickness in the retina after anti-VEGF treatment [29]. These studies suggested that HRF might have a pivotal role in retinal neuroinflammation diseases. Moreover, some preliminary studies have shown that HRF are correlated with cytokines levels in the vitreous and aqueous humor; yet, further studies are required to confirm these findings [7, 30].

Hsia et al. [31] compared the change of HRF in different retinal layers to predict visual outcomes after anti-VEGF therapy using spectral-domain optical coherence tomography (SD-OCT). They found a faster decrease of HRF in the subretinal fluid (SRF) after anti-VEGF therapy, which suggested that HRF might be used as a potential biomarker to predict the visual outcome. Our result was consistent with the above study. We found that the HRF was mainly distributed in the outer retina in patients with neovascular AMD, and the number of HRF was reduced after anti-VEGF treatment. However, there were some differences between these two studies. For example, different instruments were employed to study the HRF distribution and quantitation in different retinal layers. Hsia et al. used SD-OCT to quantify HRF in the inner retina, outer retina, and subretinal fluid layer, while we applied OCTA to characterize the HRF distribution in SCP, DCP, and outer retina. In addition, we compared the relationship between HRF change and the retinal microcapillary density, which is important for assessing the treatment outcome for AMD patients, as neovascular AMD is characterized by macular neovascularization. Hsia et al. found that the eyes with decreased HRF in the SRF after 3 consecutive anti-VEGF treatments might have better visual acuity at 12 months, suggesting that HRF might be used as a biomarker for predicting the effects of anti-VEGF treatment. In our study, we only observed the effect of anti-VEGF therapy after the three-month injections. The small sample size and relatively short-term observation are the major limitations of this study.

## 6. Conclusion

The present study suggested that HRF could be used as an indicator or sensitive biomarker for retinal inflammation in neovascular AMD. After anti-VEGF treatment, the reduced HRF number might indicate the alleviation of inflammation in the retina and, possibly, improvement in the visual function in patients with neovascular AMD. Yet, long-term observation and a study with a larger sample size should be performed to compare the relationship among HRF, visual outcome, intraretinal cysts, and similar.

## Figures and Tables

**Table 1:** Baseline characteristics of patients with neovascular AMD and comparation of HRF in the SCP, DCP, and outer retina using OCTA between baseline and after the intravitreal anti-VEGF injections.

	M0	M3	*P*
Eyes (no.)	25	—	—
Sex (male/female)	15/10	—	—
Ages (years)	71.4 ± 9.66	—	—
BCVA (logMAR)	0.84 ± 0.41	0.57 ± 0.37	<0.01^∗^
HRF-SCP (no.)	5.80 ± 1.63	5.32 ± 1.95	0.12
HRF-DCP (no.)	7.52 ± 3.06	3.76 ± 1.48	<0.01^∗^
HRF-outer retina (no.)	12.04 ± 4.91	5.88 ± 3.32	<0.01^∗^

Data are shown as a number or mean ± standard deviation. ^∗^Significant at *P* < 0.05. M0, baseline; M3, 3 consecutive monthly anti-VEGF injections. BCVA: best-corrected visual acuity; DCP: deep retinal capillary plexus; HRF: hyperreflective foci; Log MAR: the logarithm of the minimum angle of resolution; No. : number; SCP: superficial retinal capillary plexus; VEGF: vascular endothelial growth factor.

**Table 2:** Comparison of microvascular density in the SCP and DCP using OCTA between baseline and after the intravitreal anti-VEGF injections.

	M0	M3	*P*
SCP			
Fovea	17.22 ± 7.10	15.24 ± 7.78	0.10
Parafovea	43.02 ± 5.90	42.03 ± 5.78	0.32
Whole	40.40 ± 6.11	39.59 ± 5.94	0.44
DCP			
Fovea	26.85 ± 8.50	25.84 ± 8.24	0.58
Parafovea	44.42 ± 6.17	47.08 ± 5.99	0.08
Whole	42.96 ± 5.48	44.52 ± 4.58	0.24

Data are shown as mean ± standard deviation. ^∗^Significant at *P* < 0.05. M0, baseline; M3, 3 consecutive monthly anti-VEGF injections. DCP: deep retinal capillary plexus; HRF: hyperreflective foci; SCP: superficial retinal capillary plexus; VEGF: vascular endothelial growth factor.

## Data Availability

The data used to support the findings of this study are available from the corresponding author upon request.
